# Comparison of the effectiveness between transcutaneous electrical nerve stimulation, manual acupuncture, and electroacupuncture on tinnitus: study protocol for a randomized controlled trial

**DOI:** 10.1186/s13063-018-2738-9

**Published:** 2018-06-27

**Authors:** Young-Kyun Moon, Min Hee Kim, Hae Jeong Nam

**Affiliations:** 1 Department of Ophthalmology and Otolaryngology of Korean Medicine, College of Korean Medicine, Kyung Hee University, Seoul, Republic of Korea; 2 Department of Ophthalmology, Otolaryngology and Dermatology of Korean Medicine, College of Korean Medicine, Kyung Hee University Hospital at Gangdong, Seoul, Republic of Korea

**Keywords:** Tinnitus, Transcutaneous nerve stimulation (TENS), Electroacupuncture, Manual acupuncture

## Abstract

**Background:**

Transcutaneous electrical nerve stimulation (TENS) involves a neuromodulatory effect using electrical stimulation and has been widely used due to its safety and convenience. It has been used for treating tinnitus for decades. Acupuncture has also been used for tinnitus and several research studies have shown that acupuncture can improve a certain kind of tinnitus by stimulating the somatosensory system. Moreover, several studies have shown the efficacy of electroacupuncture, which is a combination of acupuncture and electrical stimulation, for tinnitus. However, the comparative effectiveness of TENS, manual acupuncture, and electroacupuncture for the treatment of tinnitus has not been determined previously. Herein, we design a randomized, non-blind clinical trial to investigate and compare the effects and safety of TENS, manual acupuncture, and electroacupuncture for tinnitus.

**Methods:**

After screening, 45 patients are randomly assigned to three groups: (1) patients in the TENS group are treated at four sites (tender points of masseter and the sternocleidomastoid muscle, in front of tragus, and mastoid process); (2) the manual acupuncture group patients are treated at 11 acupoints (TE21, SI19, GB2, TE22, ST7, TE17, GB20 of tinnitus affected side, and GB20, TE05, KI3 of both sides); (3) electroacupuncture group patients are treated by using acupuncture as in the manual acupuncture group and electrical stimulation at TE21, SI19, TE17, and GB20. Patients are treated for ten sessions, twice a week. The primary outcome measurement is the change of Tinnitus Handicap Inventory (THI) score between visit 1 and visit 10. The secondary outcome measurements are the response rate of THI, change in visual analogue scale associated with the loudness and annoyance of tinnitus, pure-tone audiometry and speech discrimination, and changes in parameters of heart rate variability.

**Discussion:**

The purpose of this study is to compare the effect of TENS, manual acupuncture, and electroacupuncture in the auricular area on tinnitus. If the specific treatment shows a significant effect compared to other treatments, it could have potential for use in clinical practice as a primary treatment.

**Trial registration:**

Clinical Research Information Service (CRIS), KCT0002117. Registered October 21, 2016. Retrospectively registered.

**Electronic supplementary material:**

The online version of this article (10.1186/s13063-018-2738-9) contains supplementary material, which is available to authorized users.

## Background

Tinnitus is a symptom in which some kind of sound is recognized in the absence of external auditory stimulation. The results of many studies indicate that 5 to 15% of the population show symptoms of tinnitus that are not alleviated by treatment. Moreover, 1 to 3% of the population with tinnitus have mental stress, sleep disorders, and reduced work productivity [1].

Treatment to relieve tinnitus symptoms include cognitive behavioral therapy [2], counseling treatment [3], tinnitus retraining therapy (TRT) [4], hearing aids [5], cochlear implant therapy [6], drug therapy [7], and various types of invasive or non-invasive electrical stimulation, such as transcranial magnetic stimulation (TMS), transcranial direct current stimulation (tDCS), and transcutaneous electrical nerve stimulation (TENS) [8]. Because there is no recommended medicine for tinnitus [9], physiotherapy is considered to have a sufficient effect, despite the lack of evidence for its long-lasting effect on tinnitus [10–12]. Among the modalities of physiotherapy, TENS is one of the most safe, non-invasive, and effective treatments [8]. Therefore, TENS has been used for treating tinnitus for decades [13–15].

In many cases of TENS treatment for tinnitus, the C2 dermatome or the vagus nerve around the ear is treated [8, 16–18]. In contrast, systemic and periauricular acupoints have been determined as the manual acupuncture (MA) and electroacupuncture (EA) treatment sites for tinnitus, according to the meridian system theory in TCM [19].

TENS is a relatively simple procedure by which electrical stimulation is applied using a quantified machine, whereas MA and EA are relatively inaccessible for those unfamiliar with TCM theory since the practitioner’s knowledge and technique are needed. However, the similarity among TENS, MA, and EA treatments for tinnitus is that the targeted treatment area is around the ear. Identification of a significant difference between the effects of physical therapy administered with TENS and MA and EA treatments in this study would help to determine the most appropriate physiotherapy stimuli and strengths for the treatment of tinnitus.

Several studies have compared the efficacy of TENS [15, 16], MA [12, 20], and EA [21] with a placebo-controlled group; in addition, one study has compared the effectiveness between MA and EA treatment for tinnitus [22]. However, trials that compared the effect of TENS and MA or TENS and EA have not been conducted. Moreover, no study has compared the effects of TENS, MA, and EA on tinnitus at the same time.

In this trial, participants are treated with periauricular TENS, systemic and periauricular MA according to the meridian system theory in TCM, and EA with MA plus periauricular electro-stimulation.

From the results of this study, we will determine which treatment is more effective for tinnitus between MA and EA with MA plus periauricular electro-stimulation. Then, the efficacy of TENS, MA, and EA will be compared to determine the most effective treatment for tinnitus.

The results of this study are expected to help in determining the first-line physiotherapy for tinnitus.

## Methods/design

### Study design

**Fig. 1:**
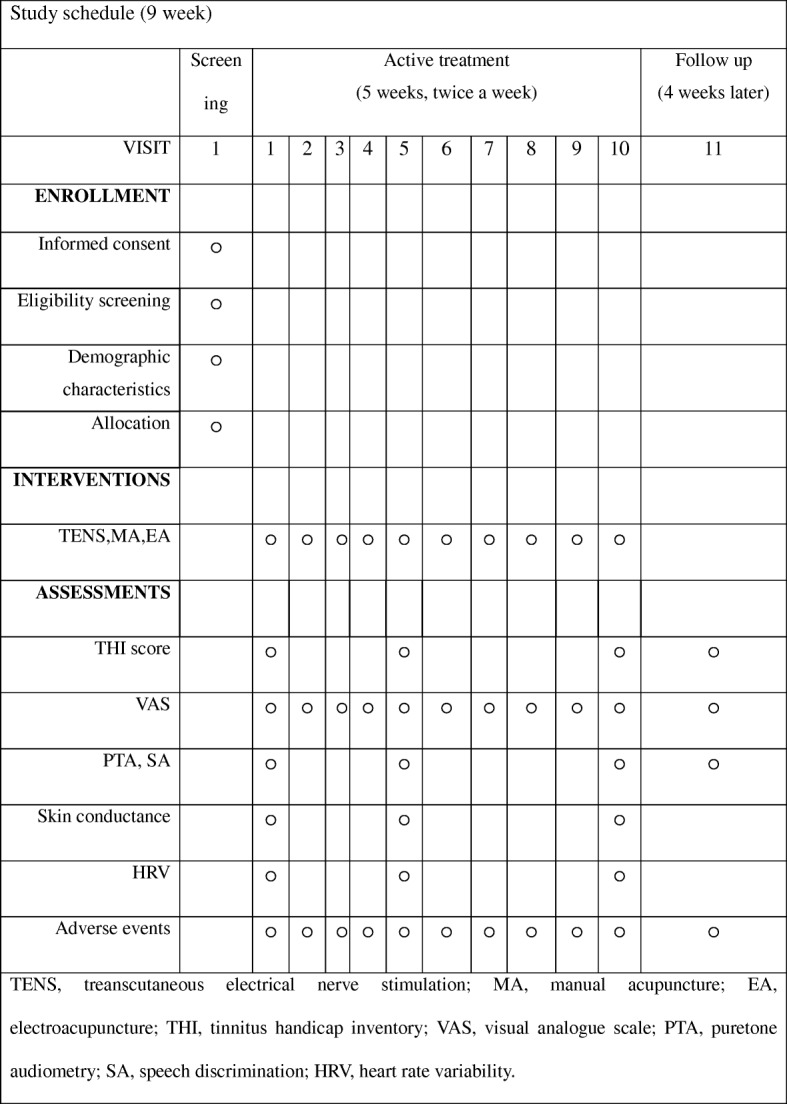
Treatment schedule

**Fig. 2:**
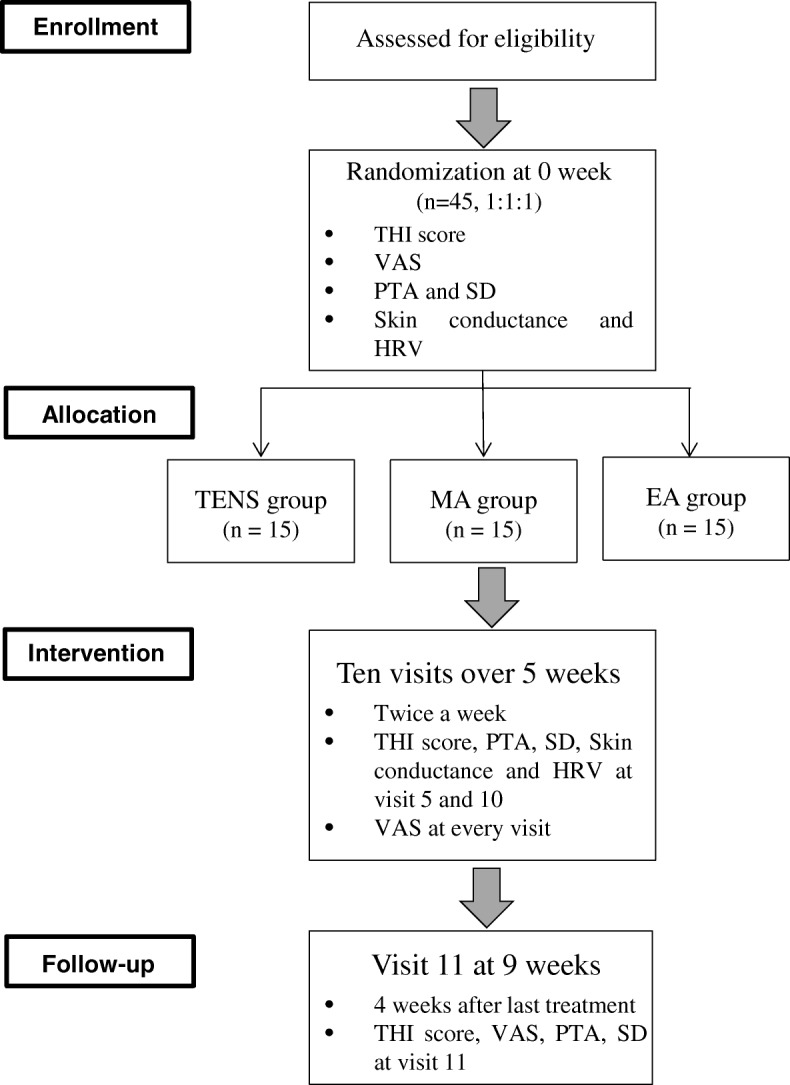
Study design flow chart. *THI* tinnitus handicap inventory, *VAS* visual analogue scale, *PTA* pure tone audiometry, *SD* speech discrimination, *HRV* heart rate variability, *TENS* transcutaneous electrical nerve stimulation, *MA* manual acupuncture, *EA* electroacupuncture

This article includes all components as described in the SPIRIT checklist (Additional file 1). This prospective, randomized, paralleled, open-labeled pilot trial is to be conducted at the Kyung Hee University Korean Medicine Hospital (Seoul, Korea). After screening, 45 participants who meet the inclusion and exclusion criteria are randomly assigned to TENS, MA, and EA groups (*n* = 15 each). After randomization, participants are treated for ten sessions, twice a week. A follow-up is conducted 4 weeks after the end of treatment. The treatment schedule and the study flow chart are shown in Figs. 1 and 2.

In the TENS group, the TENS treatment at the tender point of the sternomastoid muscle and mastoid process (C2 dermatome) is on the affected ear-side alone. In both the MA and EA groups, manual systemic acupuncture treatment is on both sides of the body and periauricular acupuncture and electro-stimulation are on the affected ear-side alone. If the participant has bilateral tinnitus, the louder tinnitus ear-side is selected. If a participant has bilateral tinnitus and feels the same loudness in both ears, right-handed patients are treated for the right-ear and left-handed patients the left-ear.

### Sample size

This trial is designed as a pilot study. The appropriate sample size of the two-arm or three-arm pilot study is reported as > 12 [23, 24]. In order to set each group to ≥ 12 participants, it is necessary to assign at least 15 participants to each group (45 in total), considering a dropout rate of 20%.

Therefore, considering the dropout rate and the characteristics of a pilot study, we set the sample size of 15 per group.

### Recruitment

Participants are recruited by two strategies. First, posters that contain brief information on the inclusion and exclusion criteria, purpose of the study, and intervention are displayed in the Kyung Hee University Korean Medicine Hospital. Second, advertisements are placed on the hospital website. The clinical trial is to be performed from June 2016 to December 2017.

### Inclusion and exclusion criteria

#### Inclusion criteria

Participants are recruited based on the following conditions:

(1) Age 20 to 75 years, either sex

(2) Presence of tinnitus symptom for more than 6 consecutive months

(3) Not under any type of medical treatment for tinnitus during the entire clinical trial period

(4) Voluntarily signed informed-consent forms

#### Exclusion criteria

Participants are excluded under any of the following conditions:

(1) Those who have received TENS or acupuncture treatment within 3 months

(2) Those who have had medicines and surgical procedures due to cardiac disorder, or patients with pacemaker implants

(3) Those who have a disease condition that can clearly cause tinnitus such as Meniere’s disease, otitis media, and otosclerosis

(4) Those with metal allergies and needle phobia

(5) Those who take psychotropic drugs to treat tinnitus symptoms

(6) Those who gave birth within 6 months, or pregnant or lactating women

(7) Those who are unable to perform evaluation and complete the questionnaire

(8) Those who are considered unsuitable for the study based on the opinion of the investigators

### Randomization and blinding

Randomization is conducted by a balanced block randomization using an R package program (version 3.2.5.). After the participants complete the screening process, they are randomly assigned to one of the groups in a 1:1:1 ratio by researchers who are not involved in the data collection. Once the group is randomly assigned, changes cannot be made.

The randomization results are open to the participant and the therapist but hidden from the assessor. It is difficult to blind the participant or the therapist because the interventions are clearly identified. The researcher who conducts the randomization will not contact the assessor. Therefore, in this study only the assessor is blinded.

### Interventions

**Fig. 3:**
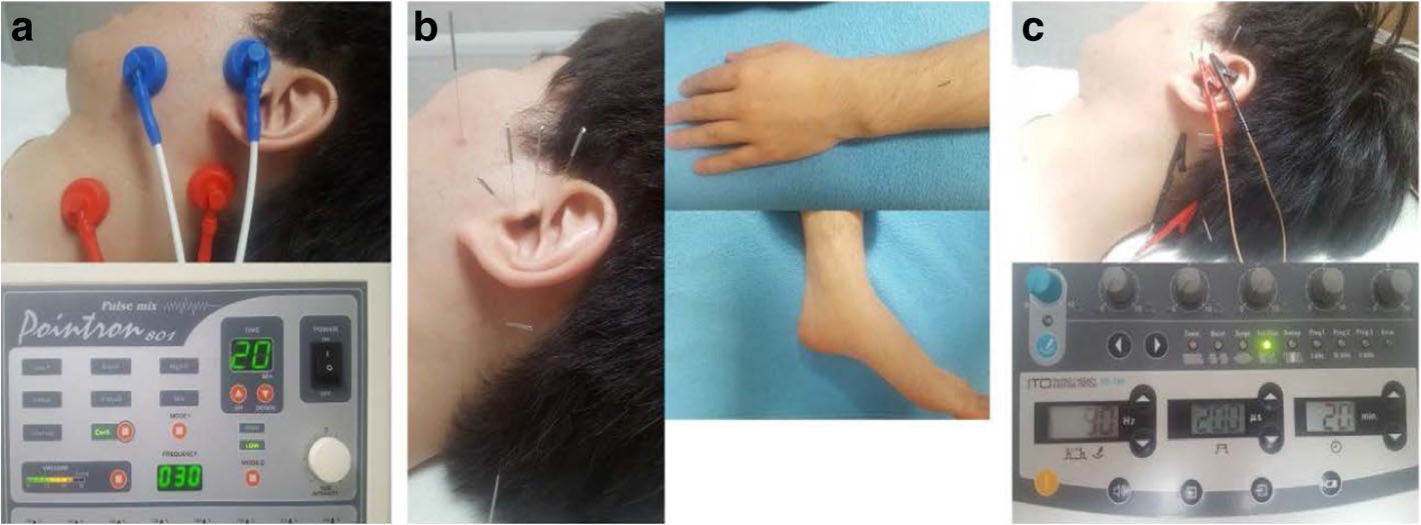
Pictures of each treatment and electrical stimulation devices. **a** Electrode attachment sites in the TENS (transcutaneous electrical nerve stimulation) group. **b** The acupoints of MA (manual acupuncture) group (TE21, SI19, GB2, TE22, ST7, TE17, GB20 of affected side, and GB20, TE05, KI3 of both sides). **c** Treatment sites of the EA (electroacupuncture) group (11 acupoints of MA group plus electrical stimulation at TE21, SI19, TE17, and GB20)

Pictures of each intervention are presented in Fig. 3.

#### Tens

TENS treatment is performed by using a low frequency electrical stimulator (Pointron 801 made by Dae Yang Medical Co., Ltd, Wonju, Korea). A pair of electrodes is attached to the tender point of the sternomastoid muscle and mastoid process (C2 dermatome) on the affected side, and the other pair of electrodes is placed at a trigger point of the masseter muscle and the temporomandibular joint in front of the tragus. Subsequently, 30 Hz electrical stimulation is applied for 20 min. The stimulation is adjusted to relatively high intensity, but is neither unpleasant nor painful.

#### Manual acupuncture

Participants in the MA and EA groups are treated using acupuncture at 11 acupoints (TE21, SI19, GB2, TE22, ST7, TE17, GB20 of the affected side, and GB20, TE05, KI3 of both sides) using a sterilized disposable stainless needle of 0.25 mm diameter and 40 mm length (Dongbang Medical Co., Boryeong, Korea). The acupuncture point was selected based on previous clinical studies [22, 25]. Needling depth is approximately 5–10 mm based on the differences in anatomical structure of the participants and the individual characteristics of acupoints. The acupuncture needles are inserted until the participant feels “De-qi”. Acupuncture needles are retained for 20 min.

#### Electroacupuncture

Acupuncture treatment for the EA group is as in the MA group. After treatment by acupuncture, the inserted needles at TE21, SI19, TE17, and GB20 are connected to an electroacupuncture stimulator (ES-160 made by Ito Co., Ltd, Tokyo, Japan) and stimulated by a mixed frequency of 30/90 Hz with 3 s intervals. Stimulation intensity is set at a relatively high level that is acceptable to the participants.

### Primary outcome measures

#### Tinnitus Handicap Inventory score

The primary outcome measure is the change of Tinnitus Handicap Inventory (THI) score between visit 1 and visit 10. THI consists of 25 questions. Each question is answered by “yes” (scored as four points), “sometimes” (scored as two points), or “no” (scored as zero points). The total score can be categorized into the following five categories: slight (0 to 16 points), mild (18 to 36 points), moderate (38 to 56 points), severe (58 to 76 points), and catastrophic (78 to 100 points). In addition, it can be categorized into four subscales: functional subscale (11 questionnaire), emotional subscale (9 questionnaire), and catastrophic subscale (5 questionnaire).

The THI questionnaire is completed at visits 1, 5, and 10 and the follow-up visit, which is 4 weeks after the end of treatment. If the total THI score at visit 10 improves by > 10 points compared with baseline, the patient is considered a “responder”; if the improvement of the total THI score is < 10 points (or worsens), the patient is considered a “non-responder”.

### Secondary outcome measures

#### THI score response rate

In addition to changes in total and each subscale score of the THI after visit 10, the response rate of the THI score between visits 1 and 5 and visits 1 and 11 is calculated.

#### Visual analogue scale

The loudness and annoyance of tinnitus are measured using the visual analogue scale (VAS). Symptoms are rated on a scale of 0 as no symptom and 10 as the greatest discomfort. When participants place a mark on the 10-cm horizontal line, the score is evaluated by measuring the distance from zero to the mark. VAS is assessed at every visit, both before and after the treatment.

#### Pure-tone audiometry and speech discrimination

Pure-tone audiometry (PTA) and speech discrimination (SD) are performed at visits 1, 5, and 10 and the follow-up session by using a clinical audiometer (GSI 61™ Audiometer, Grason-Stadler, Minnesota, USA).

#### Skin conductance and heart rate variability

Skin conductance and heart rate variability (HRV) are measured and analyzed by the PowerLab data acquisition device (PL3516, developed by ADInstruments, Dunedin, New Zealand). Skin conductance and HRV are conducted at visits 1, 5, and 10.

### Safety outcomes

Any adverse events that occur during the study period are documented in the case report form (CRF). Vital signs are checked and recorded at the screening visit.

### Statistical analysis

All collected data are analyzed using the statistical software SPSS v. 19.0 for Windows (SPSS Inc., Chicago, IL, USA). The statistical significance level is set at 5%. Continuous variables are reported as means and standard deviations, and categorical variables are reported by using frequencies and percentages.

In the case of continuous variables, if the normality test results follow a normal distribution, data are analyzed by analysis of variance (ANOVA). When the parametric test is impossible, the Kruskal-Wallis test is used. Categorical variables are analyzed by the Chi-square test or Fisher’s exact test.

Since the primary outcome is the change of THI score and the second outcome is the response rate of the THI score, THI measurements are needed at least twice or more. The second THI assessment is conducted at visit 5; therefore, the modified intention-to-treat (mITT) analysis is conducted on the patients who have completed at least five treatment sessions. The “last observation carried forward method (LOCF)” is used for missing values.

### Participant protections and ethics

This study protocol has been approved by the institutional review board (IRB) of Kyung Hee University Korean Medicine Hospital (KOMCIRB-160321-HR-012). Also, this trial has been registered at Clinical Research Information Service (CRIS, registered number KCT0002117). The trial conforms to the principles of the Declaration of Helsinki.

Participants are notified about replaceable treatments, responsibilities during the study, and the potential advantages and dangers associated with this research. Possible adverse effects caused by interventions include pain, redness, subcutaneous hemorrhage, or numbness at the acupuncture sites. However, these symptoms mostly disappear spontaneously within a few days. In the event of serious adverse effects, appropriate treatment is provided as soon as possible. All the reported symptoms are documented. Participants are discontinued from the study if the treatments cause aggravation of symptoms.

## Discussion

The pathophysiology of tinnitus is difficult to delineate because of its complexity. Hyperactivity of the auditory system caused by peripheral denervation and deprivation of auditory input is one of the potent inductive mechanisms of sensorineural tinnitus [26, 27]. Some research studies have shown that muscular problems involving the temporomandibular joint or cervical region induce somatosensory tinnitus [28, 29].

Non-invasive neuromodulation includes TMS, tDCS, TENS, and neurofeedback. Among these, TENS is relatively affordable, easy to use, and has almost no adverse effects [30].

In previous studies applying TENS for tinnitus, electrodes were attached at the TMJ, earflaps, tragus, and C2 dermatome; the results indicate response rates from 17.9 to 82% [8, 15, 20, 31–33]. The electrical stimulation of the C2 dermatome is known to control the excitement and inhibition of the dorsal cochlear nucleus through targeting cells in the dorsal column nucleus [27]. We selected the TENS treatment sites based on the above-mentioned hypotheses.

In traditional Chinese medicine (TCM), both manual acupuncture (MA) and electroacupuncture (EA) are the most frequently used physiotherapy for tinnitus. EA involves treatment with MA with the enhancement of the treatment effect by adding electrical stimulation to specific points [34]. Although none of these are considered as a primary treatment, several lines of clinical research reported the efficacy of MA and EA for tinnitus treatment [19, 21, 22]. A recent systematic review that used meta-analysis indicated that acupuncture may offer subjective benefit to patients with tinnitus [19]. Although it is difficult to understand the mechanism of acupuncture for tinnitus, it reportedly involves somatosensory stimulation [31]. Also, acupuncture might influence the function of the olivocochlear nucleus [22]. We propose to ascertain the effects of acupuncture by treating the acupoints, such as TE21, SI19, and GB2, which have been frequently used in previous studies [19, 25].

In EA, electrical stimulation is added to acupuncture. Wang et al. [22] have reported that EA has some promising short-term effects compared to manual acupuncture and placebo acupuncture. Doi et al. [21] have reported that the VAS and THI score of an EA treatment group showed significant improvement compared with the no-treatment group. However, a systematic review published in 2016 indicated that there is no convincing experimental evidence for EA as an effective treatment for tinnitus due to insufficient quality data [32]. Thus, the effect of EA on tinnitus remains controversial and requires further research. Therefore, EA is included as one of the interventions in this study.

In our previous study on tinnitus, the manual acupuncture group and distal electroacupuncture group showed a significant effect on VAS_uncomfortable_ compared with the periauricular electroacupuncture group without systemic manual acupuncture [33], possibly due to the systemic acupuncture effect of the meridian system, such as the modulation of the autonomic nervous system and balancing of body and mind. In this study, to clarify the effect of TENS, MA, and EA on the autonomic nervous system, we will examine the skin conductance and HRV.

The purpose of this study is to compare the effect of TENS, MA, and EA in the auricular area on tinnitus. If the specific treatment shows a significant effect compared to other treatments, it could have potential for use in clinical practice as a primary treatment. In contrast, if none of the treatments show more significant effectiveness than the others, the results could be used as basic data to facilitate the use of a more non-invasive therapy, such as TENS, as the first-line treatment, following the non-inferiority test for TENS.

Our study has several limitations. First, it is a non-blind trial; therefore, psychological factors of the therapist and subjects can cause bias. Second, primary outcome measurements are a subjective assessment since an assessor cannot directly detect the tinnitus symptom. To compensate for this problem, we will use the THI and VAS, the most common and credible assessments for tinnitus [10]. Third, there is no placebo-controlled group and all three interventions are active controls; hence, the significance of the effects can only be investigated through before-and-after comparison. To compensate for this problem, a change of THI score, the total THI score at visit 10 improves by > 10 points compared with baseline, is set as the primary outcome measure. Finally, this study is a pilot study to be conducted on a small sample. Additional studies will be necessary to confirm and utilize the results of the study.

### Trial status

This trial is currently recruiting participants.

## Additional file

Additional file 1: Standard Protocol Items: Recommendations for Interventional Trials (SPIRIT) checklist. The document represents the Spirit Checklist. (DOC 129 kb)

## Abbreviations

ANOVA: Analysis of variance
CRIS: Clinical Research Information Service
EA: Electroacupuncture
HRV: Heart rate variability
IRB: Institutional review board
LOCF: Last observation carried forward method
MA: Manual acupuncture
mITT: Modified intention-to-treat
PTA: Pure tone average
SD: Speech discrimination
TCM: Traditional Chinese medicine
tDCS: Transcranial direct current stimulation
TENS: Transcutaneous electrical nerve stimulation
THI: Tinnitus Handicap Inventory
TMS: Transcranial magnetic stimulation
TRT: Tinnitus retraining therapy
VAS: Visual analogue scale
